# Colorectal Cancer after Kidney Transplantation: A Screening Colonoscopy Case-Control Study

**DOI:** 10.3390/biomedicines9080937

**Published:** 2021-08-02

**Authors:** Francesca Privitera, Rossella Gioco, Alba Ilari Civit, Daniela Corona, Simone Cremona, Lidia Puzzo, Salvatore Costa, Giuseppe Trama, Flavia Mauceri, Aurelio Cardella, Giuseppe Sangiorgio, Riccardo Nania, Pierfrancesco Veroux, Massimiliano Veroux

**Affiliations:** 1 General Surgery, University Hospital of Catania, 95123 Catania, Italy; f.privitera05@gmail.com (F.P.); rossellagioco1992@gmail.com (R.G.); alba.ilari@gmail.com (A.I.C.); Cremona.simon@gmail.com (S.C.); salvatore.costa@policlinico.unict.it (S.C.); 2 Department of Biomedical and Biotechnological Sciences, University of Catania, 95123 Catania, Italy; coronadany@libero.it; 3 Pathology Unit, Department of Medical and Surgical Sciences and Advanced Technologies, University of Catania, 95123 Catania, Italy; lipuzzo@unict.it; 4 Gastroenterology Unit, University Hospital of Catania, 95123 Catania, Italy; gtrama@unict.it; 5 Faculty of Medicine, University of Catania, 95123 Catania, Italy; mauceriflavia@gmail.com (F.M.); aureliocardella@tiscali.it (A.C.); 6 Department of General Surgery and Medical-Surgical Specialties, University of Catania, 95123 Catania, Italy; pep.sang7@gmail.com (G.S.); ric.nania@gmail.com (R.N.); 7 Organ Transplant Unit, University Hospital of Catania Department of Medical and Surgical Sciences and Advanced Technologies, 95123 Catania, Italy; pveroux@unict.it

**Keywords:** kidney transplantation, post-transplant cancer, colorectal cancer, colonoscopy, screening, healthy, adenoma, cancer

## Abstract

The incidence of colorectal cancer in kidney transplant recipients has been previously reported with conflicting results. In this study, we investigated if the incidence of colorectal advanced neoplasms in kidney transplant recipients, evaluated with screening colonoscopy, was higher than in healthy individuals. One-hundred sixty kidney transplant recipients undergoing screening colonoscopy were compared with 594 age- and sex-matched healthy individuals. Advanced colorectal neoplasia was found in 22 patients (13.7%), including four patients (2.5%) with colorectal cancer. Compared with the healthy population, kidney transplant recipients did not have an increased risk of developing a colorectal cancer (OR 0.69; 95% CI 0.236–2.063, *p* = 0.688) although it developed at a younger age. In contrast, kidney transplant recipients had a higher risk of developing an advanced adenoma compared with the control group (OR 1.65; 95% CI 0.930–2.981, *p* = 0.04). In conclusion, kidney transplant recipients did not have an increased incidence of colorectal cancer compared with healthy population. However, transplant patients displayed a higher incidence of colorectal adenomas, suggesting that screening colonoscopy in kidney transplant recipients should be expanded to include even younger recipients (<50 years old).

## 1. Introduction

Kidney transplantation is the best replacement therapy for patients with end-stage renal disease [1]. The improvements of surgical techniques and newer immunosuppressive protocols led to the dramatic improvement of both short- and long-term outcomes of kidney transplantation leading to the year 2000 [2]. However, over the last three decades, short-term improvement of kidney graft survival decreased significantly, while long-term improvement remained stable, due to the wider use of older and marginal donors and, mainly, to the chronic effect of immunosuppression, which may increase the risk of cardiovascular diseases, infections, and post-transplant cancers [2].

Kidney transplant recipients are approximately three-times more likely to develop cancers than the general population [3,4,5,6,7,8]. This excess risk is greatest for oncogenic virus-linked cancers, such as Kaposi’s sarcoma, and for nonmelanocytic and melanocytic skin cancer [3,4,5,6,7,8]. For other solid-organ cancers, such as colorectal and lung cancers, the risk is increased by approximately two to three times when compared with the general population [3,4,5,6,7,8]. 

The survival among transplant recipients with advanced-stage cancer is extremely poor. Surgical treatments and intensive chemotherapy are often limited by comorbidities and potential nephrotoxicity, and the efficacy of the treatment is limited by the chronic immunosuppression, which, in most cases, is not reduced for the fear of acute graft rejection and graft loss. In this setting, the clinical screening protocols may aid, in principle, to detect early stage diseases allowing for curative treatment, finally resulting in reduced cancer-related morbidity and mortality [9,10,11,12].

Colorectal cancer is the third most commonly diagnosed cancer worldwide [13], and survival in kidney transplant recipients may be worse than in the general population regardless of the stage of diagnosis [3,4,6]. There are many studies suggesting that the incidence of colorectal neoplasia, including advanced adenomas, is significantly higher after kidney transplantation [6,10,14]; however, the data are conflicting [3,4,5,6,7,8]. Screening with guaiac-based faecal occult blood tests (FOBT) or faecal immunochemical tests (FIT) followed by colonoscopy in people over the age of 50 with average risk has been shown to reduce the mortality of colorectal cancer in the general population [15,16,17,18,19].

The Kidney Disease Improving Global Outcomes (KDIGO) clinical practice guidelines for the care of kidney transplant recipients suggested screening from the age of 50 years with annual faecal haemoglobin and flexible sigmoidoscopy screening every 5 years in all renal transplant recipients [9,10,11,12,20,21] and that this might be cost effective [22,23]. However, there are limited data on the prevalence of advanced colorectal neoplasia in kidney transplant recipients [9,10], and screening might be limited by the low efficacy of FOBT due to the expected increase of false-positives due to the toxicity of immunosuppression, cytomegalovirus infection, and minor mucosal inflammation [24,25,26].

In this study, we evaluated the efficacy and safety of a screening program, using both a faecal immunochemical test and colonoscopy to determine the prevalence and characteristics of advanced colorectal neoplasia in kidney transplant recipients compared with healthy individuals. We, therefore, evaluated the diagnostic accuracy of faecal immunochemical test for human haemoglobin to predict advanced colorectal carcinoma in this high-risk population.

## 2. Materials and Methods

### 2.1. Study Population

This was a population-based study among kidney transplantation recipients receiving a screening colonoscopy between January 2014 and October 2017 performed at a single institution in a large university hospital.

Kidney transplant recipients were eligible for this study if they had a negative pre-transplant colonoscopy, were aged over 35 years, had a functioning graft and were at least four years after transplantation. Exclusion criteria included a previous history of colorectal cancer, known or suspected familial colorectal cancer syndrome (patients with cancer in at least one close relative), chronic inflammatory bowel disease, and bleeding disorders (active bleeding). Patients receiving anticoagulant therapy were included in the study provided that they discontinued the treatment at least one week before the procedure.

Patients were invited to participate during the course of routine follow-up in the outpatient clinic. A total of 195 patients were initially screened and asked to participate in the study. Thirty-five patients were excluded from the study: 18 patients due to suspected familial colorectal cancer syndrome, 4 for bleeding disorders, and 13 did not give consent to participate. A total of 160 patients fulfilled the criteria and were finally considered for analysis.

Kidney transplant recipients received a standard three-drug immunosuppressive therapy, with or without induction therapy with anti-interleukin-2 receptor antibodies (Simulect, Novartis, Basel, Switzerland) or with antithymocyte globulin (ATG-Fresenius, Fresenius, Bad Homburg, Germany), based on both the donor and recipient characteristics as previously described [27].

Clinical and follow-up data were retrieved from our electronic database and included age, sex, body mass index, cause of end-stage renal disease, type of dialysis before transplantation, and waiting time before transplantation.

### 2.2. Screening Methodology

Study participants completed a faecal immunochemical test (FIT) for human haemoglobin using the Citest (Citest Diagnostics, Canada). This test uses a brush to obtain each faecal sample, which is then applied to a testing card, where there is an anti-Hb antibody. The FIT was repeated after one week. In the case of discrepancy between the two tests, FIT was considered positive.

After faecal testing, and regardless of the result, the participants were referred for colonoscopy. Experienced gastroenterologists performed all colonoscopies. Colonoscopy was considered complete with endoscope insertion of the caecum, which was confirmed by the identification of the ileocecal valve. Participants received bowel preparation, according to the endoscopist’s preference, with a polyethylene glycol-based preparation the day before the procedure.

All transplant patients underwent an evaluation of graft function with serum creatinine before and after colonoscopy. All abnormal lesions detected during the colonoscopy were biopsied, and biopsy forceps were used to evaluate the size of each polyp.

Advanced colorectal adenoma was defined as the presence of a tubular adenoma of at least 10 mm of diameter, a villous or tubulovillous adenoma (defined by the presence of at least 25% villous), or an adenoma with high grade dysplasia, carcinoma in situ, or intramucosal carcinoma [9,10,14,28,29]. Non-advanced neoplasia/adenoma were defined as adenomas <10 mm in diameter with low grade dysplasia and/or containing <25% villous components, while inflammatory of hyperplastic polyps were considered as normal [9,10,14,28,29]. In patients with multiple lesions, only the lesion with the highest grade was considered for the analysis.

A case-control study was performed to determine whether kidney transplant recipients had a higher prevalence of advanced colonic neoplasm. We randomly assigned two or more age- and sex-matched asymptomatic individuals who had undergone a screening colonoscopy for colorectal cancer from January 2014 to September 2017 at the gastroenterology unit, which performed the colonoscopy even in transplant recipients. Patients with previous colorectal surgery, a known history of colorectal cancer or inflammatory bowel disease were excluded from the analysis. Age matches were within 5 years and outcomes were assessed by comparing the prevalence of advanced neoplasms between the two groups.

The study was conducted in accordance with the principles of the 1975 Declaration of Helsinki, and the Ethical Committee of the University Hospital of Catania ruled that no formal ethical approval was required in this particular case, as it conformed to normal clinical practice. All patients signed an informed consent detailing all the procedures.

### 2.3. Statistical Analysis

The results and patients characteristics are reported as the raw values and percentages for the categorical data and as the mean ± standard deviation (SD). To evaluate the diagnostic accuracy of faecal haemoglobin to detect advanced colorectal neoplasia, we compared the results with the endoscopic findings by using two by two tables and the calculated sensitivity, specificity, and positive and negative predictive likelihood ratio [8,9]. Comparison of the means and percentages between patients who developed an advanced adenoma/colorectal cancer and patients with normal colonoscopic findings was estimated by the unpaired two Student’s t-test or Mann-Whitney U test, as appropriate. 

To compare the neoplastic risk in patients undergoing kidney transplantation with that of control group, standardized incidence rates (SIR) were used. The SIR was obtained by dividing the number of observed tumour cases (advanced colorectal adenoma and colorectal cancer) by the number of cases expected in the control group. The time to detect advanced colorectal adenoma and cancer after transplantation was calculated using Kaplan–Meier cumulative curves and estimated using the log-rank test. The odds ratios (OR) are reported with 95% confidence intervals (95% CI) and *p* values. A *p* value < 0.05 was considered as statistically significant.

## 3. Results

A total of 160 patients undergoing kidney transplantation between January 2000 and December 2014 were enrolled in this study. The demographical and clinical characteristics of the patients are outlined in Table 1.

One hundred forty-five patients received a kidney from a deceased donor, while 15 patients received a living donor: 8 patients received a dual kidney transplantation, 7 a simultaneous kidney-pancreas transplantation, and 15 a second transplant. Among these patients, 145 (90.6%) received a screening colonoscopy after transplantation, and 15 (9.4%) received a diagnostic colonoscopy. The mean age was 49.2 years, and the mean interval from transplantation to colonoscopic examination was 6.4 ± 2.1 years. Glomerulonephritis and autosomal polycystic kidney disease were the most common causes of ESRD, while most patients were on haemodialysis as pre-transplant renal replacement therapy with a mean time of pre-transplant dialysis of 41.3 ± 39.5 months. Colonoscopy with intubation of the caecum was completed in all cases; however, in four cases, the colonoscopy was repeated due to incomplete bowel preparation.

**Figure 1:**
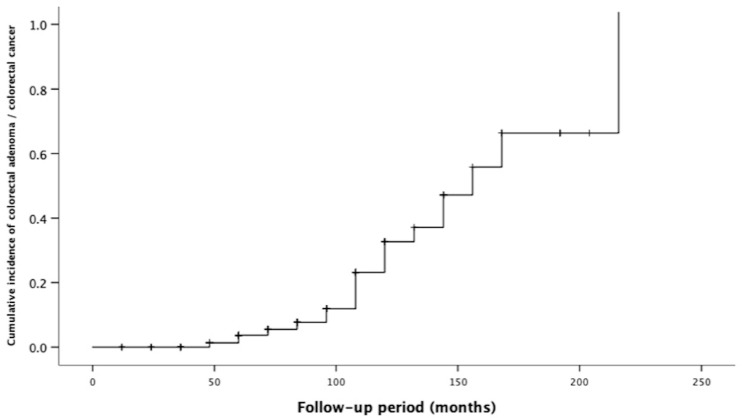
Kaplan–Meier analysis on the cumulative risk of overall colorectal adenoma according to the time from kidney transplantation.

Overall, 22 patients (13.7%) had advanced colorectal neoplasia (Table 2), and the incidence of advanced colorectal neoplasia increased with the time from transplant (Figure 1).

Four patients (2.5%) had a high-grade dysplastic lesion and underwent an endoscopic resection (two patients) or a colonic resection (two patients). Four patients (2.5%) had a previously undiagnosed colorectal cancer: one patient had an advanced stage colon cancer at the time of diagnosis (T4N2M1) and died two months after diagnosis, while the other three patients (two T1N0M0 and one T2N0M0) underwent an R0 colonic resection and were alive at the last follow-up. The remaining 14 patients with villous adenoma underwent a complete endoscopic resection of the colorectal neoplasia. Data on the staging of the cancers are detailed in Table 2.

There was no significant difference in the baseline characteristics between patients with a positive colonoscopy and those who did not have advanced colorectal cancer; however, patients with advanced colorectal neoplasia were older and had a significantly longer follow-up, suggesting a role of long-term immunosuppression in the development of colorectal cancer. The faecal immunochemical test was positive in 114 patients (71.2%) (Table 3).

The positive likelihood ratio for FIT in detecting advanced colorectal neoplasia was 1.3 (95% CI 1.10–1.5), while the negative likelihood ratio was 0.2 (95% CI 0.07–1.13). The overall sensitivity and specificity of FIT were 90.9% and 30.8%, respectively. Indeed, non-neoplastic disease was frequently detected at colonoscopy: diverticular disease (n = 27, 16.8%) and haemorrhoids (n = 12, 7, 5%) were the most common diagnoses; however, surprisingly, 19 patients (11.8%) presented with a chronic inflammatory disease (5 with Crohn’s disease, 4 with ulcerative colitis, and 9 with mycophenolate mofetil-related colitis).

Colonoscopy was completed without significant adverse events in all patients. One patient had a minor bleeding after a polypectomy, which was treated conservatively. There was no significant change in the serum creatinine before and after the colonoscopy in all patients, and all patients returned to their normal social activities the day after the procedure.

All 18 patients with advanced colorectal adenoma completed the 1-year follow-up colonoscopy, without evidence of recurrence. All patients were alive at the median follow up of 4.5 years, while two patients lost the graft during the follow up due to chronic rejection.

We identified 594 age- and sex-matched controls who had a screening colonoscopy. FIT positivity (70%), weight loss (14.1%), and abdominal pain (12.1%) were the most common indications for colonoscopy in symptomatic patients. The control group consisted of 288 females (48.4%) and 306 males (51.6%), with a mean age of 51.1 ± 12 years. There were no significant differences in age and sex compared to the study group (*p* = 0.723 in age; *p* = 0.822 in sex). The findings of colorectal lesions at colonoscopy are reported in Table 2. When compared with the control group, the kidney transplant recipients had a significantly higher incidence of drug-induced colitis as well as de novo inflammatory bowel disease (*p* < 0.05). 

The analysis of control group revealed that 63 (10.6%) patients had a colorectal neoplasia: of these, 21 (3.5%) patients had colorectal cancer, and 42 (7%) patients had an advanced adenoma. Patients in the control group with colorectal cancer were most frequently male (52.4%) with a mean age of 66.4 ± 11.2 years. Patients with advanced colorectal neoplasm were more frequently female (54.7%) with a mean age of 65.7 ± 12.3 years. 

When compared with transplant patients, the control group patients with colorectal cancer (66.4 ± 11.2 vs. 54.2 ± 9.5 years, *p* < 0.01), or with advanced adenoma (65.7 ± 12.3 vs. 59.3 ± 8.7 years, *p* < 0.05) were significantly older at the time of diagnosis. Kidney transplant recipients had a similar risk of developing a colorectal cancer compared with the age- and sex-matched control group (OR 0.69; 95% CI 0.236–2.063, *p* = 0.688, SIR 0.70; 95% CI 0.324–1.895) although it developed at a younger age. In contrast, kidney transplant recipients had a higher risk of developing an advanced adenoma compared with the control group (OR 1.65; 95% CI 0.930–2.981, *p* = 0.04, SIR 1.60; 95% CI 1.01–2.785) (Table 4).

## 4. Discussion

This study showed that screening colonoscopy could be useful to reduce the incidence of advanced colorectal cancer in kidney transplant recipients. The incidence of colorectal cancer in kidney transplant recipients has been previously reported with conflicting results. In the study of Park et al. [14], the incidence of colorectal adenoma among 315 kidney transplant patients was 22.9%, while 6 patients (1.9%) developed cancer. 

When compared to healthy subjects undergoing a colonoscopy for colorectal cancer surveillance, kidney transplant recipients had an OR of advanced adenoma of 3.52 (95% CI, 1.90–6.53) and an even greater risk of developing cancer compared with the control subjects (OR, 12.0; CI, 1.45–99.7; *p* = 0.021) [14]. Similar results were reported by Hall et al. [7], who reported a higher 5-year cumulative incidence of colorectal cancer in kidney transplant recipients compared with that of US general population at the recommended age of screening.

A recent study [30], investigating the incidence of colorectal cancer (CRC) among haemodialysis patients undergoing kidney transplantation found increased odds of 1.34 of the cumulative incidence of CRC in those undergoing kidney transplantation compared with patients on haemodialysis not undergoing kidney transplantation. However, other recent studies did not confirm such findings and reported a similar incidence of colorectal cancer between transplant patients and the general population [4,6].

This study confirmed this assumption: among the 160 patients undergoing a colonoscopy, 22 (13.7%) developed an advanced colorectal neoplasia, including 4 (2.5%) patients with colorectal cancer. When comparing the incidence of colorectal cancer in healthy individuals (21/595, 3.5%), kidney transplant recipients were not at increased risk of developing a colorectal cancer. In contrast, the incidence of advanced adenoma in kidney transplant recipients (18/160, 11.2%) was significantly higher than in healthy individuals (42/595, 7%, *p* = 0.04). This apparent dichotomy was probably explained by the earlier time of colonoscopy: in their study, Kwon et al. [10] demonstrated that the incidence of colorectal neoplasm in previously pre-transplant negative patients, increased with the time from transplant, reaching 21.1% after 10 years.

Many risk factors have been proposed for the development of colorectal cancer in kidney transplant recipients. Immunosuppression may impair host immunity to tumorigenesis and allow reactivation of oncogenic viruses [31,32]. Long-term exposure to calcineurin inhibitors is considered the most important risk factor for the development of post-transplant malignancies [8,10], likely promoting tumour growth by causing the mutation responsible for the adenoma-to-carcinoma transition [10,33]. 

This was confirmed by our study: advanced adenoma developed more frequently in male patients who were older than 50 years and with more than 5 years of transplantation, thus, suggesting that long-term exposure to immunosuppressive therapy may have a role in the progression of colorectal adenoma. However, when compared with healthy individuals, kidney transplant recipients with colorectal cancer or with advanced adenoma were significantly younger at the time of diagnosis, suggesting that screening programs should be extended also to recipients <50 years.

Four kidney transplant recipients (2.5%) developed colorectal cancer in this study. One patient was diagnosed at an advanced stage and died two months after diagnosis, while, in three asymptomatic patients, the screening colonoscopy allowed for an early diagnosis, and they all underwent a radical surgical resection with no sign of recurrence at the last follow up.

Mortality for colorectal cancer in kidney transplant recipients is higher than in the general population and increases over time [29,34,35,36], and most patients present with advanced stage at diagnosis [37]. This raises the need for timely and appropriate screening programs, which may allow an early diagnosis at a time when the cancer is potentially curable.

Colonoscopy surveillance might be an appropriate approach in transplant population. In patients with chronic kidney disease, FIT appears to be an accurate screening test, such that a negative test may rule out the diagnosis of colorectal cancer within 2 years. However, the risk of major complications from work-up colonoscopy may be at least ten-fold higher than in the general population [34]. 

In the first study on colonoscopy screening of kidney transplant recipients, Collins et al. [9] evaluated the diagnostic accuracy of FIT and the prevalence of colorectal adenoma in the transplant population: 29 patients (13%) were diagnosed with an advanced neoplasia, of whom 5 (2%) had colorectal cancer, suggesting a higher prevalence in the transplant population. Interestingly, FIT showed a low sensitivity and a reasonable specificity. Similar results were reported by Kwon et al. [10], who reported an incidence of 8.1% of advanced colonic neoplasm, including a 1.6% of patients presenting with colorectal cancer, with an increased odds of advanced colonic neoplasm of 2.3-times greater than the general population.

In asymptomatic patients, FIT could be able to correctly rule out CRC and avoid colonoscopy in 75–80% of non-transplant patients [38,39]. In our study, FIT was positive in 71.2% of kidney transplant patients. The overall sensitivity and specificity of FIT were 90.9% and 30.8%, respectively, suggesting that FIT could be a reliable screening method for gastrointestinal disease in kidney transplant recipients although, in most cases, a benign disease is found at colonoscopy. Our study confirmed the low specificity of FIT, likely as a consequence of the high rate of colonic inflammatory disease and non-specific colic inflammation occurring in the renal transplant population [24,25,26]. Notably, this study reported a high prevalence of unrecognized inflammatory bowel disease (11.9%), with nine patients presenting a de novo IBD.

This study might be limited by the relatively small sample size: however, this study presented a homogenous cohort of kidney transplant recipients and healthy population, thereby, eliminating the potential confusing factors, such as differences in races, immunosuppression, and follow up. Moreover, all colonoscopies were performed at a single institution. We were not able to match the control group for the comorbid conditions affecting the transplant population: however, the only characteristic associated with an increased risk of colorectal adenoma/cancer in the transplant patients was age. Not all patients undergoing kidney transplantation in the study period performed a screening colonoscopy, and this might underestimate the incidence of colorectal cancer in this population.

Despite these potential limitations, this study presents some improvements upon previous studies. The incidence of advanced colorectal neoplasia in kidney transplant recipients was higher than in the general population, and it presented at a younger age compared to the general population. This additional evidence reinforces the suggestion to provide screening colonoscopy even in kidney transplant recipients younger than 50 years old.

## 5. Conclusions

In this colonoscopy study, we found that kidney transplant recipients were at increased risk of developing an advanced colorectal adenoma and, potentially, a colorectal cancer compared to the general population. Kidney transplant recipients should be considered as a high-risk population for the development of colorectal cancer at a younger age compared to the general population, and thus colonoscopy screening should be offered to transplant patients younger than 50 years old to increase the likelihood of detecting the colorectal neoplasia at an earlier and potentially curable stage, thereby, improving the long-term outcomes. A prospective study with a large sample size could be useful to better evaluate the risk of colorectal cancer in kidney transplant recipients.

## Figures and Tables

**Table 1:** The clinical data of 160 kidney transplant recipients undergoing post-transplant colonoscopy. Patients with advanced colorectal neoplasia (22 patients) were compared with those without colorectal neoplasia (138 patients).

Characteristics	Entire Study Group	Advanced Colorectal Adenoma	Negative Findings	*p* Value
N	160	22	138	
Deceased/living donor	145/15	18/4	127/11	0.825
Age (years)	49.2 ± 8.15	57.3 ± 8.77	52.6 ± 10.21	<0.05
Sex (M/F)	95/65	13/9	82/56	0.228
BMI (Kg/m^2^)	25.9 ± 12.2	26.4 ± 3.8	25.7 ± 4	0.473
Cause of End-stage Renal disease (n, %)				
Polycystic kidney disease	40 (25)	6 (27.3)	34 (24.7)	0.895
Glomerulonephritis	62 (38.7)	11 (50)	51 (36.9)	0.334
Diabetes mellitus	5 (3.1)	1 (4.5)	4 (2.9)	0.324
Others *	30 (18.9)	2 (9.1)	28 (20.3)	0.222
Unknown	23 (14.3)	2 (9.1)	21 (15.2)	0.332
Waiting List (months)	20.2 ± 10.5	18.5 ± 15.6	19.4 ± 21.6	0.775
Pre-transplant dialysis (months)	41.3 ± 39.5	34.7 ± 21.7	35.3 ± 40.2	0.545
Haemodialysis/peritoneal dialysis	129/16	15/5	114/11	0.645
No dialysis (n, %)	15 (5)	2 (9)	13 (9.2)	0.876
Dual kidney transplantation (n, %)	8 (5)	2 (9)	6 (4.2)	0.777
Second transplantation (n, %)	15 (9.3)	3 (15)	12 (8.5)	0.634
Kidney-pancreas transplant (n, %)	7 (4.3)	0 (0)	7 (5)	0.231
Donor age (years)	47.7 ± 15.8	50.8 ± 13.8	52.5 ± 11.1	0.325
Donor terminal serum creatinine (mg/dL)	1.24 ± 0.6	1.22 ± 0.7	1.28 ± 0.7	0.654
Cold ischemia time (min)	918 ± 335.5	921 ± 321.8	932 ± 355.5	0.644
Immunosuppression (n, %)				
Tac/MMF/Ster	120 (75)	14 (63.7)	105 (76)	0.735
CyA/Ever/Ster	12 (7.5)	2 (9)	10 (7.3)	0.865
Tac/Ever/Ster	12 (7.5)	4 (18.3)	9 (6.5)	0.745
Ever/MMF/Ster	6 (3.8)	0 (0%)	6 (4.4)	0.553
CyA/MMF/Ster	10 (6.2)	2 (9)	8 (5.8)	0.622
Time to transplant (years)	6.5 ± 8.4	8.1 ± 7.7	6.8 ± 4.1	<0.01
Mean serum creatinine	1.90 ± 1.11	1.89 ± 1.13	1.77 ± 1.31	0.432
Significant Comorbidities (n, %)				
Diabetes	13 (8.1)	3 (13.6)	10 (7.2)	0.0308
Cardiovascular disease	22 (13.7%)	4 (18.1%)	18 (13)	0.515
Chronic lung disease	6 (3.7)	1 (4.5)	5 (3.6)	0.832

BMI: body mass index; TAC: tacrolimus; MMF: mycophenolate mofetil; STER: steroids; CyA: cyclosporine; and Ever: everolimus. * Others included reflux uropathy, IgA-nephropathy, Alport’s syndrome, and membrano-proliferative disease.

**Table 2:** The colonoscopic findings in kidney transplant recipients compared with the healthy controls.

	Transplant Recipients (N = 160) (%)	Healthy Controls (N = 594)	*p*
Colonoscopic Findings			
Colorectal cancer	4 (2.5)	21 (3.5)	0.535
High-grade adenoma	4 (2.5)	8 (1.3)	0.093
Tubulovillous, villous adenoma	10 (6.2)	22 (3.7)	*p* < 0.05
High-grade tubular adenoma > 10 mm	4 (2.5)	12 (2)	0.735
Low-grade tubular adenoma	5 (3.1)	42 (7)	0.723
Hyperplastic polyp	8 (5)	50 (8.4)	0.735
Ulcerative colitis	4 (2.5)	5 (0.8)	*p* < 0.05
Crohn disease	5 (3.1)	4 (0.6)	*p* < 0.05
Mycophenolate mofetil- colitis	9 (5.6)	0 (0)	*p* < 0.01
Other (Sarcoma di Kaposi, Pseudomembranous colitis)	3 (1.8)	0 (0)	*p* < 0.01
Diverticulosis	20 (12.7)	87 (14.6)	0.732
Haemorrhoids/Normal Findings	84 (52.5)	343 (57.7)	0.331

**Table 3:** The diagnostic accuracy of the faecal immunochemical test for the detection of advanced colorectal neoplasia in kidney transplant recipients.

Advanced Colorectal Neoplasia	Positive	Negative	Total
Present	20	2	22
Absent	96	42	140
Total	116	44	160

**Table 4:** The incidence of colorectal neoplasm between kidney transplant recipients and a healthy control.

	Kidney Transplant Recipients (n = 160)	Control Group (n = 594)	Odds Ratio (95% CI)	*p* Value
Advanced Adenoma (%)	18 (11.2)	42 (7)	OR 1.65 (0.930–2.981)	0.04
Colorectal cancer (%)	4 (2.5)	21 (3.5)	OR 0.69 (0.236–2.063)	0.688

## Data Availability

The data are restricted by Centro Nazionale Trapianti because of ethical issues and to protect donor and recipient identities. It is possible for de-identified data to be made available upon reasonable request.

## Abbreviations

FOBT	faecal occult blood tests
FIT	faecal immunochemical test
KDIGO	Kidney Disease Improving Global Outcomes
CRC	colorectal cancer
BMI	body mass index
TAC	tacrolimus
MMF	mycophenolate mofetil
STER	steroids
CyA	cyclosporine
Ever	everolimus
IBD	inflammatory bowel disease
